# E-Wallet: A Study on Cashless Transactions Among University Students

**DOI:** 10.12688/f1000research.73545.1

**Published:** 2022-06-21

**Authors:** Anushia Chelvarayan, Sook Fern Yeo, Han Hui Yi, Hazlaili Hashim

**Affiliations:** 1 Faculty of Business, Multimedia University, Melaka, Malaysia; 2 Department of Business Administration, Daffodil International University, Dhaka, Bangladesh

**Keywords:** E-Wallet, Intention, University Students, Perceived usefulness, Perceived ease of use, perceived risk, trust

## Abstract

E-wallet is an application that enable users to download payment cards using a mobile device. It is a new trend for consumers to use an e-wallet application to replace the traditional payment method. With E-wallet, a user does not need to bring cash or a credit card along with them. It enables users to make purchases in a more convenient way. Hence, this research analyses the factors that affect university students’ intention to use e-wallet. The Technology Acceptance Model (TAM) serves as the theory underpinning this research A total of 140 respondents from a Malaysian private institution participated in this study. Convenience sampling was used to select samples, and respondents completed the questionnaire using a Google form and a paper and pencil approach. The questionnaire was created using a nominal scale and a five-point Likert scale. Descriptive analysis, reliability analysis, and multiple regression analyses were utilised to analyse the data in this study. Students, supervisors, academics, researchers, learning institutions, commercial organisations, and the government will all benefit immensely from the data and information gathered from this study as we will be able to examine and understand the factors that influence students’ decision to use an e-Wallet for their daily financial operations. This study, however, has certain limitations as it does not reflect the complete student population in Malaysian tertiary education and only examines four variables: perceived usefulness, perceived ease of use, perceived risk, and trust. Future studies could focus on other impacting elements such as risk, complexity, pervasive technology use and tech-savvy future generations.

## Introduction

An e-wallet is a mobile application that allows users to download payment cards. The usage of an e-wallet application to substitute a traditional payment method is a new trend among customers. A user does not need to bring cash or a credit card while using an e-wallet. It allows people to acquire items that meet their requirements and desires in a more convenient manner. Due to the advancement of technology, there are many e-wallet platforms that exist in the market such as Touch n Go, Boost and Grabpay. Hence, this research will analyse the factors that affect university students’ intention to use an e-wallet by adopting the Technology Acceptance Model (TAM).

Previous research carried out by Yap & Ng (2019), claimed that convenience, confidentiality and social influence are the contributing factors for people to use an e-wallet ^1^ application. Users believe that an e-wallet brings a lot of convenience in their life as technology helps users to complete their tasks in a faster and convenient way. This study will use perceived usefulness (PU), perceived ease of use (PEOU), perceived risk (PR) and trust (T) to measure the acceptance of e-wallet among university students in Malaysia.

## Literature review

E-wallet is an application that enables user to use it when making purchases. According to Qasim, Siddiqui & Rehman (2012), e-wallet is a mobile application that enables the consumers to make financial transactions. ^2^

Technology Acceptance Model (TAM) is partly adopted in this research to determine the intention of consumers using e-wallet. Besides TAM, there are another two variables that will be used in identifying the intention for consumers to use e-wallet, which are perceived risk (PR) and trust (T). This is to ensure that readers can clearly observe the relationship with all the independent variables on the intention of university students to use e-wallet. TAM was developed by Davis in 1989 and it was developed based on a previous theoretical model called Theory of Reasoned Action (TRA). ^3^

E-wallet is a new market trend which has been widely used by everyone. It brings many benefits to users as it offers quite a number of services. Therefore, it is important for researchers to know users’ intention in using e-wallet so that e-wallet platform developers can improve to serve users better. According to Zhao et al. (2010), intention is a person’s willingness and eagerness to obtain something desirable. ^4^ Additionally, Venkatesh et al. (2003) stated that intention to use can be defined as the consumer’s interest and desire to try the new product and services. ^5^ In this context, Tan et al. (2020) stated that the key variables of TAM which are perceived usefulness (PU), and perceived ease of use (PEOU) are the key determinants of using a new technology intentionally. ^6^

In this study, there are four independent variables that will be adopted in determining the intention to use e-wallet. The independent variables are perceived usefulness (PU), perceived ease of use (PEOU), perceived risk (PR) and trust (T).

In the technology acceptance model (TAM), perceived usefulness (PU) is one of the most commonly used variables and the most important component to examine the impact towards the adoption of e-wallet. Perceived usefulness (PU) refers to the level of belief perceived by the consumers when they are using the new tools to do their tasks. ^7^ Perceived usefulness can be said to be a strong indicator to determine the user acceptance towards technology. ^7^

Another significant component in the technology acceptance model (TAM) is the perceived ease of use (PEOU), where it measures the convenience and how easy for users to use e-wallet in their daily life. Davis (1989) defined perceived ease of use (PEOU) as a person’s thought on how easy to use a new technology or system. ^3^ According to Davis, Bagozzi & Warshaw (1989), the degree of easiness in terms of physical and mental on using a new technology perceived by a person is the perceived ease of use (PEOU). ^7^ From here, the contributing factor of PEOU is significant enough to affect user’s intention to use a new technology, in this study, the e-wallet. Based on previous research done by Guriting & Ndubisi (2006), there is a significant positive relationship between perceived ease of use (PEOU) and consumer intention to use e-wallet. ^8^

Perceived risk (PR) on the other hand is the customer feelings of predictability of an unfavourable outcome. ^9^ According to Widodo, Irawan & Sukmono (2019), perceived risk (PR) is the possible losses assumed by the consumers when they are purchasing goods. ^10^ The examples of potential losses are monetary losses, privacy breach, safety issues, bad user experience and waste of time. ^10^ According to Razif, Misiran, Sapiri & Yusof (2020), consumers will hesitate when they want to purchase a good, especially expensive goods. ^11^ Consumers might worry that the goods purchased does not meet their expectation. By using e-wallet, there are certain risks faced by the users such as leaking of personal information to unauthorized parties. Therefore, this might cause worry among users.

Trust can be defined as the level of consumer belief on the value and usefulness of e-wallet services. ^12^ According to Malik & Annuar (2019), trust is described as a person’s feelings toward another person in completing a task, and it is a critical aspect for electronic monetary services. ^13^ Trust would result in better achievement while doubt results in failure that will stop people from further using online payment systems. ^14^^,^^15^ Trust is an important independent variable to investigate the adoption of technology. Based on previous research done by Ajmera & Bhatt (2020), trust is a significant aspect to decide the consumer’s intention towards a particular technology. ^16^

## Methods

**Figure 1.**
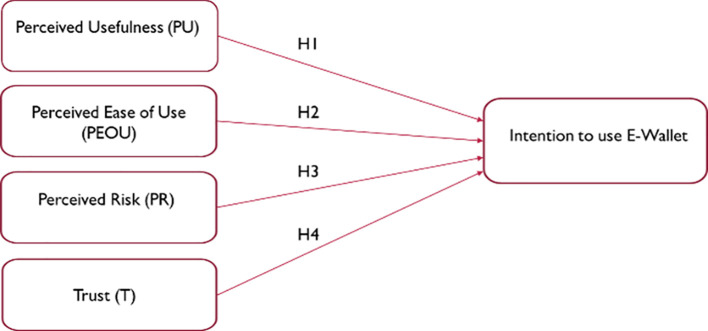
Theoretical framework (Developed for this research).

The research adopted two variables from the Technology Acceptance Model (TAM) and another two variables: perceived risk (PR) and trust (T). The following research framework is developed for this study:

The link between the dependent and independent variables is depicted in Figure 1. The research framework was built around the links between intention to use e-wallet as the dependent variable and perceived usefulness (PU), perceived ease of use (PEOU), perceived risk (PR) and trust (T), as the independent variables. In this research especially during the COVID – 19 pandemic, the independent variables are employed as intermediary variables to examine factors affecting e-wallet use among university students (as shown in the Underlying Data). ^20^ Consumers were spending more via online transactions as they were not able to visit physical stores due to the Movement Control Order in Malaysia This research uses the descriptive research design whereby a descriptive research design is a type of research design that aims to obtain information to systematically describe a phenomenon, situation, or population. More specifically, it helps answer the what, when, where, and how questions regarding the research problem. As of 31 March 2021, a total of 1.32 million students pursued their tertiary education in Malaysia, whereby 52.1% registered in public universities and 47.9% were in PHEIs. ^17^ Hence, a total of 140 students from a Malaysian private institution participated in this study as part of the sample population. In this research G-Power 3.1.9.7 has been used to find out the sample size. The sample size recommended by G-Power is 129. However, the sample size of this research was 140 students. Convenience sampling was used to pick samples, and respondents completed the questionnaire using a Google form and a paper and pencil approach. The questionnaire (shown in the Extended data) ^21^ which was adopted from G. M. M. Dewi *et al*. (2021) ^18^ and A,M,A Nashren *et al.* (2019), ^19^ were then distributed to the respondents and included a written statement requesting their consent before proceeding to fill in. The respondents who proceeded to answer the questionnaire automatically consented to participate as research respondents. The questionnaire was created using a nominal scale and a five-point Likert scale. Descriptive analysis, reliability analysis, and multiple regression analysis were utilised to analyse the data in this study. The demographic data, the ideas included in this study, and their interactions were all analysed using the Statistical Package for the Social Sciences (SPSS (Statistical Package for the Social Sciences) is a software program used by researchers in various disciplines for quantitative analysis of complex data. It is a software package created for the management and statistical analysis of social science data.). The processed data was double checked by team members. It went through a thorough screening process.

The study was reviewed and approved by research ethic committee, Multimedia University Malaysia. The responses of each respondent were kept confidentially. The data were collected and analyzed anonymously. Ethical approval number: EA2342021.

## Results and discussion

To provide a clear understanding, the findings are reported in the tables below. The theories that were created were also tested and summarised as follows:

Table 1 summarises the demographic information collected for this research, with a total of 140 respondents who are students from a private University in Malaysia.

**Table 1.** Demographic information of the respondents.

	Frequency (n)	Percentage (%)
**Gender**		
Female	88	62.9
Male	52	37.1
**Ethnicity**		
Malay	9	6.4
Chinese	124	88.6
Indian	6	4.3
Others	1	0.7
**Age group**		
Below 18	0	0
18 to 20	32	22.9
21 to 23	100	71.4
24 and above	8	5.7
**Level of education**		
Post-secondary (Foundation/A-Levels/STPM/Matriculation)	23	16.4
Tertiary (Diploma/Bachelor’s Degree)	115	82.1
Postgraduate (Masters/PhD)	2	1.4
Others	0	0
**Employment status**		
Full-time employment	6	4.3
Part-time employment	15	10.7
Self-employed	1	0.7
Unemployed	118	84.3
**Gross monthly income (Malaysian Ringgit – RM)**		
Less than RM1,000	127	90.7
RM1,000 – RM1,499	4	2.9
RM1,500 – RM1,999	6	4.3
RM2,000 – RM2,499	2	1.4
RM2,500 or above	1	0.7
**Number of e-wallet application(s) you have in your mobile phone currently**		
None	3	2.1
1	35	25.0
2	50	35.7
3	31	22.1
4 and above	21	15.0
**How often do you use the e-wallet application (s) for the past 1 month?**		
I never use any e-wallet application	6	4.3
I use e-wallet less than 5 times per month	81	57.9
I use e-wallet 5 to 10 times per month	44	31.4
I use e-wallet more than 10 times per month	9	6.4
**In what occasion will you use the e-wallet?**		
Payment	116	82.9
Transfer money	57	40.7
Claim government money	59	42.1
Online shopping	76	54.3

Table 2 shows the Cronbach’s Alpha for each variable. All of the variables in this study had Cronbach’s Alpha values over 0.7, indicating that they were all acceptable. The independent factors with the greatest Cronbach’s Alpha are perceived risk (0.912), trust (0.878), perceived ease of use (0.844), and perceived usefulness (0.819). At the same time, Cronbach’s Alpha for the dependent variable of intention to use E-Wallet is 0.814. Cronbach’s Alpha is used to measure the reliability of all the variables. Hence, the results in Table 2 shows that all independent variables are reliable and acceptable.

**Table 2.** Reliability analysis for all variables.

Independent variables	Cronbach’s Alpha	Number of items
Perceived Usefulness (PU)	0.819	4
Perceived Ease of Use (PEOU)	0.844	4
Perceived Risk (PR)	0.912	4
Trust (T)	0.878	4

**Table:** 

Dependent variable	Cronbach’s Alpha	Number of items
Intention to use E-Wallet	0.814	4

Table 3 shows the result of coefficients analysed by SPSS. It demonstrates the relationship between dependent and independent variables through multiple linear regression analysis. The hypotheses are supported if the p-value (significance level) does not exceed 0.05. Thus, Table 3 shows that three of the independent variables of the study i.e., perceived usefulness, perceived risk and trust have a significant positive relationship towards intention to use e-wallet among students, whereby the variables of p-value of perceived usefulness, perceived risk and trust is 0.000, 0.002 and 0.018 respectively. However, perceived ease of use does not have a significant relationship toward the intention to use e-wallet among students. This is due to the p-value is more than 0.05 as the p-value is 0.068.

**Table 3.** Coefficients of all variables.

Model	Unstandardized coefficients		Standardized coefficients	t-test	Significance level
	Beta	Standard error	Beta		
(Constant)	0.737	0.293		2.516	0.013
PU	0.370	0.085	0.353	4.337	0.000
PEOU	0.135	0.073	0.154	1.838	0.068
PR	0.166	0.052	0.236	3.196	0.002
Trust	0.184	0.077	0.194	2.394	0.018

Based on the above summary in Table 4, H1, H3 and H4 are accepted as the p-value is less than 0.05. This shows that perceived usefulness, perceived risk and trust have a significant relationship with the intention to use e-wallet among students. However, H2 is rejected because the p-value is more than 0.05. Hence, there is no significant relationship between perceived ease of use and intention to use e-wallet among students.

**Table 4.** Hypotheses summary for all variables.

No	Hypothesis	p-value	Result
**H1**	There is a significant relationship between perceived usefulness (PU) and intention to use e-wallet.	0.000	Accepted
**H2**	There is a significant relationship between perceived ease of use (PEOU) and intention to use e-wallet.	0.068	Rejected
**H3**	There is a significant relationship between perceived risk (PR) and intention to use e-wallet.	0.002	Accepted
**H4**	There is a significant relationship between trust (T) and intention to use e-wallet.	0.018	Accepted

## Discussion

The purpose of this research is to study the e-wallet acceptance among university students. The four independent variables of this research are perceived usefulness, perceived ease of use, perceived risk and trust while the dependent variable of this research is intention to use e-wallet. In order to determine the sample size, G-Power has been used to determine the accurate sample size. 140 questionnaires were distributed to the target respondents. Furthermore, the SPSS software has been used to analyze the validity of the data collected. Moreover, the relationship between the independent variables and dependent variable has been determined.

In conclusion, three independent variables of this research were found to have significant relationship with intention to use e-wallet. These three independent variables are perceived usefulness, perceived risk and trust. On the other hand, perceived ease of use does not have significant relationship with intention to use e-wallet. Hence, this research will benefit the consumers. The consumers can know more about e-wallet and realize that there are more people to using e-wallet nowadays. For those consumers who are still not using e-wallet, they will be more confident towards e-wallet after reading this research because they will realize that many people are using e-wallet these days especially during pandemic and e-wallet actually brings a lot of benefits to the consumers. In near future, the number of e-wallet users in Malaysia will increase.

## Conclusion

The research was conducted to investigate the factors affecting the intention to use e-wallet among university students especially during the COVID-19 pandemic in Malaysia. This research will influence future studies for e-wallet adoption among students in Malaysia, especially during the current global pandemic. The findings from this research could contribute to the development of e-wallet platforms especially for student market. The findings are useful for online platform or application developers whereby they can use the findings from this research to enhance their existing platforms or develop new platforms specially dedicated for students.

However, when conducting this study, there were some constraints that should be noted. To begin with, the sample size in this study was modest, with only 140 participants. As a result, the findings may not be able to provide an accurate picture of e-wallet adoption among Malaysian students.

Secondly, the data could not be collected easily in a short period of time as there were many challenges faced by the researcher. This is due to the fact that the majority of responders were unwilling or unable to complete the questionnaire as they feel it was too much effort for them and a waste of time his makes the data collection difficult for researchers.

Further research should be conducted to gather a larger sample size from students from other parts of Malaysia. To improve the accuracy of the data collected, the questionnaires should be sent to multiple locations.

Finally, this study clearly demonstrates students’ perceptions on the intentions to use e-wallet. As a result, mobile learning developers can attract more users by making mobile learning systems that are more user-friendly and publicising their benefits to students.

## Data availability

### Underlying data

Chelvarayan, AC (Multimedia University) (2021): *E-Wallet: A Study on Cashless Transactions Among University Students.* DANS [Dataset] https://doi.org/10.17026/dans-z3u-j7c8. ^20^

This project contains the following underlying data:

• E-WALLET ACCEPTANCE AMONG UNIVERSITY STUDENTS.csv. Responses from participants.

### Extended data

Chelvarayan, AC (Multimedia University) (2022): *E-Wallet: A Study on Cashless Transactions Among University Students.* DANS. [Dataset] https://doi.org/10.17026/dans-xzh-qzjc. ^21^

This project contains the following extended data:

• Questionnaire administered to participants

Data are available under the terms of the Creative Commons Zero “No rights reserved” data waiver (CC0 1.0 Public domain dedication).

## Author contributions

All authors contributed equally for this research including the overall direction, the development of the framework, the collection of data, the analysis of the data and manuscript writing.
