# Supplementary material for: E-Wallet: A Study on Cashless Transactions Among University Students
Source: F1000Res. 2022 Jun 21;11:687. [Version 1] doi: 10.12688/f1000research.73545.1 (PMC10719559; doi:10.12688/f1000research.73545.1)
Supplement: Peer review history

# Reviewer report: Reviewer response for version 1

**DOI:** 10.5256/f1000research.77201.r202820

**Published:** 2023-12-13

**Authors:** Femmy Effendy

**Affiliations:** 1 Sekolah Tinggi Manajemen Informasi dan Komputer, Karawang, Indonesia

The articles reviewed clearly and accurately cite the latest literature examining the acceptance and use of e-wallets as well as literature references from the TAM theory proposed by Davis. An appropriate research design uses quantitative descriptive and inferential methods. The details of the methods and analysis provided are sufficient to allow other parties to imitate them because they are based on clear models and theories and use measurable methods.

 Statistical analysis and interpretation are correct, but the gradual stages of analysis such as validity, reliability, normality and regression tests must be further explained to obtain the results of the relationship between variables. The conclusions drawn are sufficiently supported by the results, but need to be linked to previous research, whether the research carried out is in accordance with the reference of the referenced article or rejects the reference. so that the research results strengthen previous theories or weaken previous theories and do not matter about the sample size taken

Is the work clearly and accurately presented and does it cite the current literature?

Yes

If applicable, is the statistical analysis and its interpretation appropriate?

Yes

Are all the source data underlying the results available to ensure full reproducibility?

Yes

Is the study design appropriate and is the work technically sound?

Yes

Are the conclusions drawn adequately supported by the results?

Yes

Are sufficient details of methods and analysis provided to allow replication by others?

Yes

Reviewer Expertise:

Bussines, Digital marketing, adoption technology, marketing management, strategic management, supply chain management, system information management

I confirm that I have read this submission and believe that I have an appropriate level of expertise to confirm that it is of an acceptable scientific standard.

# Reviewer report: Reviewer response for version 1

**DOI:** 10.5256/f1000research.77201.r143478

**Published:** 2022-08-01

**Authors:** Fathey Mohammed

**Affiliations:** 1 School of Computing, Universiti Utara Malaysia (UUM), Sintok, Malaysia

The paper analyses the factors that affect university students’ intention to use e-wallets. E-wallet application use is an interesting area of research. However, this study has many major issues and it's not publishable in current format. The authors need to consider the following comments:

**General comments:**

 -A lot of studies investigated the factors influencing the use of e-wallet applications. However, this study failed to report what has been done in the literature, so the gap is not clear and accordingly the contribution is questionable. Please, refer to some of the most relevant studies as follows ^1^^,^^2^^,^^3^^,^^4^^,^^5^^,^^6^^,^^7^^,^^8^

 -What is the research problem? What is special in the use of e-wallet applications in the private educational institution targeted in this study?

 -Why is the TAM model selected to be the underpinning model? Why were “behaviour intention” and “actual use” omitted? Why were only perceived risk and trust included? Why were, for example, complexity, pervasive technology use and tech-savvy future generations not including in the current study model?

 -Integrating perceived risk and trust with TAM has been done in the literature (refer to the above references), so what is the theoretical contribution of this study?

 -The main independent variable measured in this study is “intention to use” and based on the definition “intention is a person’s willingness and eagerness to obtain something desirable”, however based on the survey only 6 respondents haven’t used any e-wallet application, while 134 respondents use e-wallet applications frequently. So, how to measure the intention to use among those who already use the application?

 -The sample size is limited. “As of 31 March 2021, a total of 1.32 million students pursued their tertiary education in Malaysia”, and the sample size is only 140, so the results cannot be generalized.

 -Poor academic writing. To me, the following are unsound statements/arguments:

- “An e-wallet is a mobile application that allows users to download payment cards.”
- “It allows people to acquire items that meet their requirements and desires in a more convenient manner”.
- “Students, supervisors, academics, researchers, learning institutions, commercial organisations, and the government will all benefit immensely from the data and information gathered from this study”
- “This is to ensure that readers can clearly observe the relationship with all the independent variables on the intention of university students to use e-wallet”
- “Therefore, it is important for researchers to know users’ intention in using e-wallet so that e-wallet platform developers can improve to serve users better.”
- “Consumers might worry that the goods purchased does not meet their expectation.”
- “This research uses the descriptive research design whereby a descriptive research design is a … ”
- “The demographic data, the ideas included in this study, and their interactions were all analysed using the Statistical Package for the Social Sciences (SPSS)”
- “The theories that were created were also tested and …”
- “This research will influence future studies for e-wallet adoption among students in Malaysia, especially during the current global pandemic.”
- “The findings are useful for online platform or application developers whereby they can use the findings from this research to enhance their existing platforms or develop new platforms specially dedicated for students.”

-The paper structure is misleading:

- The model’s constructs were introduced in the Literature Review section, while the model was presented in Methods section. Also, the hypotheses are missed. I suggest having a section “proposed model and the hypotheses” to include everything about the proposed model.
- “Results and discussion” and “Discussion” are two subsections.

**Specific Comments:**

 - **Abstract**: It lacks the problem statement and the main results. Remove the limitations from the abstract.

 - **Introduction**: In the introduction you should provide a background of the problem by introducing the research area then narrowing down to the specific problem of the current study. The problem should be supported by most recent and related literatures and reports. The motivation and significance of the study should be highlighted. Then the main aim of the study should be stated. The paper structure then may be presented (recommended).

 - **Literature review**: This section mainly presented a theoretical background related to TAM and the factors investigated in the current study, however this section should report and analyse the most related studies.

 - **Method**: The methodology is not well organized and justified. The research design should be clearly presented.

 - **Results and discussion**: Remove “discussion” from the title, you can use “Analysis and results”. In this section, all measurements and criteria should be clear and justified. Reliability was tested using Cronbach’s Alpha, but the validity of the constructs was not measured. It is not clear how was SPSS used to test the hypotheses?

 - **Discussion**: What is presented in this section is a conclusion. In this section, the results should be discussed to show how the aim was achieved and how this study contributed to the area of research in compared with similar studies’ results.

 Regards

Is the work clearly and accurately presented and does it cite the current literature?

No

If applicable, is the statistical analysis and its interpretation appropriate?

Partly

Are all the source data underlying the results available to ensure full reproducibility?

No

Is the study design appropriate and is the work technically sound?

No

Are the conclusions drawn adequately supported by the results?

Partly

Are sufficient details of methods and analysis provided to allow replication by others?

No

Reviewer Expertise:

Technology innovation adoption, information system and e-business

I confirm that I have read this submission and believe that I have an appropriate level of expertise to state that I do not consider it to be of an acceptable scientific standard, for reasons outlined above.

## References

[1] : E-wallet: factors influencing user acceptance towards cashless society in Malaysia among public universities. *Indonesian Journal of Electrical Engineering and Computer Science* .2020;20(1) : 10.11591/ijeecs.v20.i1.pp67-74 10.11591/ijeecs.v20.i1.pp67-74

[2] : Factors Influencing the Use of E-wallet as a Payment Method among Malaysian Young Adults. *Journal of International Business and Management* .2020;3(2) : 10.37227/jibm-2020-2-21 10.37227/jibm-2020-2-21

[3] : E-Wallet Acceptance among Undergraduates in Malaysia. *TEST Engineering and Management* .2020;12990-12998

[4] : E-wallet Adoption: A case in Malaysia. *International Journal of Research In Commerce and Management Studies* .2020;2(2) :216-233

[5] : Understanding the determinants of e-wallet continuance usage intention in Malaysia. *Qual Quant* .2021; 10.1007/s11135-021-01276-7 1-27 10.1007/s11135-021-01276-7 34776542 PMC8576795

[6] : Sharing economy through e-wallet: Understanding the determinants of user intention in Malaysia. *Journal of Marketing Advances and Practices* .2020;2(2) :1-18

[7] : The Effect of Perceived Usefulness, Perceived Ease of Use, Reward, and Perceived Risk toward E-Wallet Usage Intention.17: 10.1007/978-3-030-65147-3_8 115-130 10.1007/978-3-030-65147-3_8

[8] : Understanding the Factors Influences Users Continuous Intention Towards E-Wallet in Malaysia: Identifying the Gap. *Research in Management of Technology and Business* .2020;1(1) :312-325

# Reviewer report: Reviewer response for version 1

**DOI:** 10.5256/f1000research.77201.r141632

**Published:** 2022-07-07

**Authors:** Fararishah Abdul Khalid

**Affiliations:** 1 Monash Business School, Monash University, Melbourne, VIC, Australia

The paper reads well as a whole, bringing out a good topic and valuable data. There are just some minor issues that I noticed at the beginning, with regards to references. In the Introduction, the authors used Harvard style and then had a mix of numbered style and Harvard style in Literature Review.

 In the Methods section, the authors could provide a description of the sample (on what basis were they considered as a respondent for the study, how were they identified and invited to participate). Maybe talk more about the convenient sampling method that they used. The last sentence in the second paragraph of Methods section mentioned about a screening process. It may be good to expand on the screening process.

 The second paragraph of the Methods section seems more appropriate for a Theoretical Framework section. The discussion section needs to provide more interpretation of the results, for example, how does perceived usefulness influence the intention to use e-wallet, and analyse why perceived ease of use if not significant in impacting intention to use e-wallet. This could give the paper more depth in terms of its findings.

Is the work clearly and accurately presented and does it cite the current literature?

Yes

If applicable, is the statistical analysis and its interpretation appropriate?

Yes

Are all the source data underlying the results available to ensure full reproducibility?

Partly

Is the study design appropriate and is the work technically sound?

Yes

Are the conclusions drawn adequately supported by the results?

Partly

Are sufficient details of methods and analysis provided to allow replication by others?

Partly

Reviewer Expertise:

NA

I confirm that I have read this submission and believe that I have an appropriate level of expertise to confirm that it is of an acceptable scientific standard.
